# Supplementary figures and images for: Bioinformatic characterization of ENPEP, the gene encoding a potential cofactor for SARS-CoV-2 infection
Source: PLoS One. 2024 Dec 11;19(12):e0307731. doi: 10.1371/journal.pone.0307731 (PMC11633960; doi:10.1371/journal.pone.0307731)

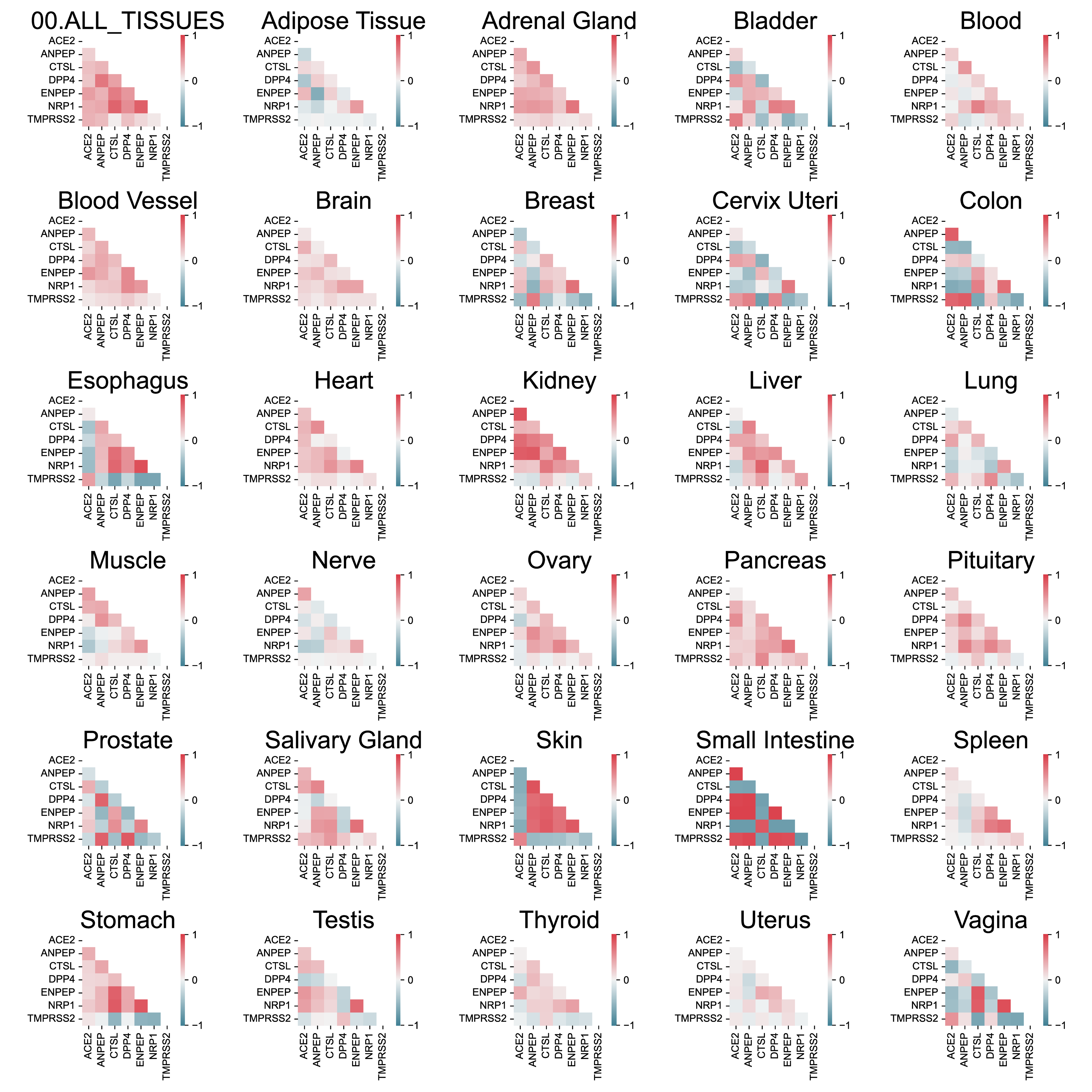

Supplement: S1 Fig — Bulk RNA-Seq expression data from the GTEx dataset (v8) [45] was retrieved as TPM values and Spearman correlation analysis was performed using the SciPy [48] Python library and heatmaps for each tissue were generated using the Seaborn [47] and Matplotlib [46] Python libraries. (TIF) [file pone.0307731.s001.tif]
