# Supplementary material for: Bioinformatic characterization of ENPEP, the gene encoding a potential cofactor for SARS-CoV-2 infection
Source: PLoS One. 2024 Dec 11;19(12):e0307731. doi: 10.1371/journal.pone.0307731 (PMC11633960; doi:10.1371/journal.pone.0307731)
Supplement: Attachment — Submitted filename: Author_Responses_final.docx [file pone.0307731.s012.docx]

REVIEWER COMMENTS AND RESPONSES

# Reviewer 1 Comment 1

**The manuscript provides a detailed bioinformatic analysis of ENPEP (encoding glutamyl aminopeptidase), focusing on its expression in various tissues and its potential role as a cofactor in SARS-CoV-2 infection. The use of large-scale publicly available datasets, including GTEx and single-cell RNA-Seq data, to assess the expression and function of ENPEP is commendable. The study explores correlations between ENPEP and ACE2, the primary receptor for SARS-CoV-2, and highlights the need for further experimental validation of ENPEP's role as a viral receptor. The manuscript is well-structured, with a clear flow of logic and detailed methodology.**

**However, there are a few areas that require clarification or improvement to strengthen the manuscript.**

## Author Response 1

We thank the reviewer for their appreciative and constructive comments on the manuscript. Below we address each point separately, and indicate all substantive changes made.

# Reviewer 1 Comment 2

**1. Rationale and Hypothesis:**

**While the manuscript clearly articulates the importance of studying ENPEP in the context of SARS-CoV-2 infection, it would benefit from a more explicit statement of the underlying hypothesis. The rationale behind choosing ENPEP as a potential cofactor for SARS-CoV-2 should be emphasized more, particularly in comparison to other cofactors like TMPRSS2 and NRP1.**

## Author Response 2

We thank the reviewer for this comment and we agree that additional statements on the central thesis of ENPEP being a gene and protein of potential interest in COVID-19 are merited. We have made the following additions to the Abstract, Introduction, and Discussion.

We have modified/added the following new text to the Abstract:

- “Recent studies have proposed a role for ENPEP as a viral receptor in humans, and ENPEP and ACE2 are both closely involved in the renin-angiotensin-aldosterone system proposed to play an important role in SARS-CoV-2 pathophysiology.”

We have modified/added the following new text to the Introduction (italics indicates old text when new text is a modification of an existing sentence):

1. “The RAAS role of APA is of particular interest, as RAA dysregulation has been proposed to play an important part in SARS-CoV-2 pathophysiology, with impaired ACE2 function being linked to some of the deleterious cardiovascular effects seen in COVID-19 such as increased pulmonary artery pressure and coagulation in swine models [(Rysz et al. 2021)](https://paperpile.com/c/aBw5Qw/AnpN). APA might be a significant contributor to these adverse effects, due sharing ANG2 as a substrate with ACE2 [(Te Riet et al. 2015)](https://paperpile.com/c/aBw5Qw/7C6R). With impaired ACE2 function, ANG2 could instead be preferentially processed by APA.”
2. “*Present in a broad range of tissues, including the small intestine, kidney cortex, liver, brain and vasculature, APA plays various general and tissue-specific roles, including activity in angiogenesis, kidney function, the endometrial cycle and implantation (L. Li et al. 1993; Marchiò et al. 2004; Velez et al. 2017; Mizutani et al. 2020), with corresponding connections to several disease processes, such as a variety of cancers, renal dysfunction****,*** preeclampsia, and possibly COVID-19 via RAAS dysregulation (Blanco et al. 2014; Chuang et al. 2017; H. Wu et al. 2023; Velez et al. 2017; Hariyama et al. 2000).**”**

We have modified/added the following new text to the Discussion:

- “Many studies have investigated the use of angiotensin receptor blockers (ARBs) for the treatment of RAAS dysregulation in COVID-19, but no conclusive evidence for the efficacy of these treatments has emerged [(Matsuzawa et al. 2022)](https://paperpile.com/c/aBw5Qw/oFho). It will be interesting to see whether the novel antihypertensive APA-inhibitor firibastat [(Khosla et al. 2022)](https://paperpile.com/c/aBw5Qw/f0SV) would counteract RAAS dysregulation in COVID-19 if the drug clears clinical trials.”

# Reviewer 1 Comment 3

**A discussion of why ENPEP, despite its relatively low expression in the lung, is of interest would add to the manuscript's depth.**

## Author Response 3

This is indeed an interesting observation generally about both ENPEP and the known SARS-CoV-2 receptor ACE2 itself, both having low levels of expression in the lung. This may also be further evidence of a more complex situation underlying COVID-19 pathology, which ultimately involves dysregulation of RAS/RAAS and infection of circulatory cells and inflammation of vasculature.

The low level of ACE2 expression in the lung has been mentioned in the original text of the Introduction:

1. “Several studies on the tissue tropism of ACE2, the primary viral receptor, found that it was rather weakly expressed in the lung, contrary to expectations based on the typical manifestation of COVID-19 as a respiratory illness (5)...”
2. “These findings are further supported by our prior study characterizing ACE2 expression based on bulk and single-cell RNA-Seq datasets, which reported low levels of ACE2 expression in the lung (9).”

The relevance of ENPEP in fibroblasts of lung has been mentioned in the original text of the Discussion:

1. “These characteristics attributed to alveolar fibroblasts are remarkably similar to those of pericytes, defined by their localization in the basal lamina directly beneath the endothelium and a characteristic morphology consisting of cytoplasmic processes [(67)](https://paperpile.com/c/fMVWwv/sjeA), suggesting that these alveolar fibroblasts might play similar roles in the lung parenchyma, guiding vessel development around the alveoli, with APA playing a regulatory role.”

We have added the following new text to the Discussion:

- “Importantly, as we and others have shown previously, expression of ACE2 in the lung is very limited, with arterial vascular endothelial cells of the lung showing the greatest proportion of ACE2 expression at just 2.55% of cells [(Barker and Parkkila 2020; Adams et al. 2020)](https://paperpile.com/c/aBw5Qw/vqF3+lQ6v). Our current analysis of scRNA-Seq data from the Human Protein Atlas data likewise shows limited expression of ACE2 in the lung, where the highest distribution of expression is observed in a type 2 alveolar cell cluster, with just 3.6% of cells expressing. By comparison, in the same dataset, ENPEP is expressed in 6.5% of lung fibroblasts and 6.5% of lung smooth muscle cells. This low level of expression of ACE2 in the primary organ of pathology in COVID-19 infection points to other functional causes of disease severity to which ENPEP may be a contributor, namely the RAS/RAAS.”

# Reviewer 1 Comment 4

**2. Methodological Clarity:**

**The bioinformatic methods used in this study are comprehensive; however, the manuscript could benefit from a more detailed explanation of certain steps, especially for readers less familiar with bioinformatics. For instance, more clarity on the thresholds used for coexpression analysis and gene ontology enrichment would be helpful. It is also important to include justification for selecting specific datasets and cutoff values in the analyses.**

## Author Response 4

We thank the reviewer for pointing this out and we endeavor to make the approaches more comprehensible to non-experts in bioinformatics. Thresholds are often set at higher levels to narrow results to specific biological functions (e.g., cell adhesion) or set more loosely to allow determination of broader biological programs (e.g., cell cycle). Depending on the dataset, thresholds can be set based on upon over-abundance or deficit of correlating elements. However, commonly a Speaman/Pearson value of 0.7–0.8 is used to denote “strong” correlation. An often cited (PMID: 23638278; 7,700+ citations) article on the topic sets the following delineations: moderate (0.50–0.70), high (0.70–0.90), very high (0.90–1.00).

As a result, we have added a reference to this article at the mention of the correlation thresholds used, in the Methods and Results sections, respectively:

- “*Genes satisfying the* high *correlation threshold cutoff (≥0.7)* [(Mukaka 2012)](https://paperpile.com/c/fMVWwv/pJEN) *and a...*”
- “*Using expression values from all tissues in the GTEx database and a* high *correlation cutoff of 0.7* [(Mukaka 2012)](https://paperpile.com/c/fMVWwv/pJEN),...”

# Reviewer 1 Comment 5

**3. Single-cell RNA-Seq Analysis:**

**The single-cell RNA-Seq data provides valuable insights, particularly regarding ENPEP expression in specific cell types like pericytes and enterocytes. However, it is recommended that the authors further elaborate on the potential functional implications of ENPEP expression in these cells, particularly in the context of viral entry and infection.**

## Author Response 5

Thank you for the comment emphasizing the need for clarity on the functional implications regarding the expression pattern of ENPEP. In the Discussion we examined several possible tissue roles for ENPEP in enterocytes and pericytes, and now have added discussion in the context of viral entry and infection of enterocytes and pericytes. We also summarized some of the main ideas near the end of the discussion.

We have modified/added the following new text to the Discussion (italics indicates old text when new text is a modification of an existing sentence):

- “While It appears that the expression profiles of ACE2 and ENPEP align in vasculature and particularly pericytes, *we found that many of the known SARS-CoV-2 cofactors had weak correlations with ACE2 in blood vessels*.“
- *“The absence of ENPEP epithelial cell expression outside the small intestine also limits the areas where APA could encounter an outside pathogen,* making it unlikely to have a role in the initial target cells of SARS-CoV-2: small intestine enterocytes appear to be the only surface cell type exhibiting significant ENPEP expression. While SARS-CoV-2 does directly infect enterocytes [(Lehmann et al. 2021)](https://paperpile.com/c/aBw5Qw/lno1), COVID-19 is nevertheless primarily a respiratory illness primarily transmitted by droplets or aerosols [(Hu et al. 2021)](https://paperpile.com/c/aBw5Qw/tpcT). While more study is required on the interplay of ACE2 and ENPEP in the intestine if APA is found to be a SARS-CoV-2 cofactor, the most interesting interactions between APA, ACE2, and SARS-CoV-2 are likely found in blood vessels, noting the overlapping expression profiles in pericytes [(Schlingemann et al. 1996; He et al. 2020; Barker and Parkkila 2020)](https://paperpile.com/c/aBw5Qw/EoNC+0bXw+vqF3) and the significance of the vascular system in COVID-19 [(Siddiqi et al. 2020; Rysz et al. 2021)](https://paperpile.com/c/aBw5Qw/rllJ+AnpN).”
- “In summary, both APA and ACE2 appear to be present in systemic vasculature and pericytes in particular — a cell type implicated to have a significant role in COVID-19 [(He et al. 2020)](https://paperpile.com/c/aBw5Qw/0bXw). If SARS-CoV-2 utilizes APA as a cofactor to infect enterocytes, this could contribute to systemic inflammation in COVID-19 and influence other pericyte-mediated functions such as capillary and blood-brain-barrier permeability [(Armulik et al. 2005; Balabanov and Dore-Duffy 1998)](https://paperpile.com/c/aBw5Qw/uSwt+bGqo). ENPEP and ACE2 both have important roles in the RAAS, with RAAS dysregulation suggested as an important part of COVID-19 pathophysiology [(Rysz et al. 2021)](https://paperpile.com/c/aBw5Qw/AnpN).”
- “*If SARS-CoV-2 interacts with APA it could help explain the vascular effects of COVID-19 since pericytes are a systemic reservoir of cells in which APA and ACE2 are more strongly correlated than many of the currently known cofactors.* This pericyte-targeted infection could promote the systemic vascular inflammation, capillary leakage, and blood-brain-barrier disruption discussed above.”

# Reviewer 1 Comment 6

**Additionally, the authors should discuss the limitations of using scRNA-Seq data, such as potential dropout effects or the challenge of distinguishing between similar cell types.**

## Author Response 6

We appreciate the comment by the reviewer as this had indeed not been addressed adequately. We have added a study limitations and future directions section at the end of the Discussion (see Author Response 7).

# Reviewer 1 Comment 7

**4. Experimental Validation:**

**While the manuscript provides compelling bioinformatic evidence for ENPEP as a potential cofactor, it would greatly benefit from experimental validation, as suggested by the authors. The authors should discuss the feasibility of experiments, such as knockdown or overexpression studies, to validate ENPEP's role in SARS-CoV-2 infection. If experimental validation is beyond the scope of this study, the authors should clearly indicate this as a limitation and suggest future directions for experimental follow-up.**

## Author Response 7

This is a very useful comment for additional considerations, as in Comment 6, we have now included together with the newly-added study limitations additional discussion on future directions.

- “The study performs several analyses based on RNA-Seq data, both bulk and single-cell. Actual levels of protein and RNA expression are often loosely correlated and thus high or low values of gene expression may not fully reflect the actual biological state of the tissues analyzed. Additionally, scRNA-Seq has inherent limitations, including low sequencing depth and dropout events. Classification of cell types by gene signatures, especially for highly specialized cells or specific states, is an evolving field which leaves room for interpretation and future improvement. As this study provides an initial bioinformatics characterization, further experimental studies are merited which directly test the role of ENPEP as a potential co-factor in SARS-CoV-2 infection, and the roles of ENPEP and ACE2 in RAS/RAAS in COVID-19 pathophysiology. For the next investigatory steps, we envision co-immunoprecipitation investigation of ENPEP with the full SARS-CoV-2 proteome and RNA-Seq of ACE2-expressing airway epithelial cell lines in ENPEP knockdown, knockout, and overexpression.”

# Reviewer 1 Comment 8

**5. Figure Presentation:**

**The figures, particularly those presenting tissue-specific expression and immunohistochemistry images, are informative. However, some figures could be improved for clarity. For example, Figure 1 presents tissue-specific ENPEP expression, but it would be helpful to include additional labeling or annotations to make it more accessible to readers.**

## Author Response 8

Thank you for the suggestion of making Figure 1 more accessible to readers.

We have made the following modifications to the Figure 1 image:

- Adjusted x-axis text for readability (size and rotation).
- Removed Ensembl identifiers.
- Updated y-axis title to better reflect content of plot.
- Added grid lines for better interpretability.

We have made the following modifications to the Figure 1 caption (italics indicates old text when new text is a modification of an existing sentence):

- “*Figure 1. Tissue specific expression of ACE2 and ENPEP* in 55 human tissues*.* Bulk RNA-Seq *expression data from the GTEx dataset (v8)*[*(45)*](https://paperpile.com/c/fMVWwv/Y2Lt) *was retrieved as TPM values and* boxplot *figures were generated using the Seaborn* [*(49)*](https://paperpile.com/c/fMVWwv/iqLI) *and Matplotlib* [*(49)*](https://paperpile.com/c/fMVWwv/pB5A) *Python libraries.* Each boxplot displays the distribution of expression values for the gene-tissue intersection: the box represents the interquartile range (IQR), the lower boundary marks the 1st quartile (Q1), the upper boundary marks the 3rd quartile (Q3), and the horizontal line within the box indicates the median. The whiskers extend to the maximum and minimum values within 1.5 times the IQR”

We have made the following modification/addition to the Figure 8 caption (italics indicates old text when new text is a modification of an existing sentence):

- “Each boxplot displays the distribution of expression values for the gene-tissue intersection: the box represents the interquartile range (IQR), the lower boundary marks the 1st quartile (Q1), the upper boundary marks the 3rd quartile (Q3), and the horizontal line within the box indicates the median. The whiskers extend to the maximum and minimum values within 1.5 times the IQR.”

We have made the following modification/addition to the Figure 9 caption (italics indicates old text when new text is a modification of an existing sentence):

- “Each boxplot displays the distribution of expression values for the gene-tissue intersection: the box represents the interquartile range (IQR), the lower boundary marks the 1st quartile (Q1), the upper boundary marks the 3rd quartile (Q3), and the horizontal line within the box indicates the median. The whiskers extend to the maximum and minimum values within 1.5 times the IQR.”

# Reviewer 1 Comment 9

**The immunohistochemistry images should also be clearly labeled with magnification and staining details for better interpretation.**

## Author Response 9

Thank you for these suggestions for improvement of Figures 2 and 3.

We have made the following modifications to the Figure 2 image:

- Image staining details enhanced.
- Insert added to panel C of Bowman’s capsule epithelium.
- Alveolar cells indicated in panel E.
- Magnification bars added to each panel: corresponding to 100 µm.

We have made the following modifications to the Figure 2 caption (italics indicates old text when new text is a modification of an existing sentence):

- “*The positive signal in the glomerulus shows a typical podocyte-type staining pattern, and the signal is also present in Bowman´s capsule epithelium* (arrowheads in insert).”
- “*In the lung, positive staining is localized in* alveolar cells (a)”
- “Bars 100 µm.”

We have made the following modifications to the Figure 3 image:

- Image staining details enhanced.
- Alveolar cells indicated in panel A.
- Magnification bars added to each panel: corresponding to 100 µm.

We have made the following additions to the Figure 3 caption:

- “Adipocytes (a) are negative for immunostaining.”
- “Bars 100 µm.”

# Reviewer 1 Comment 10

**6. Age and Sex-related Differences:**

**The analysis of age and sex-related differences in ENPEP expression is an interesting aspect of the study. However, the biological significance of these findings should be further discussed. How do these differences relate to known age and sex-related differences in COVID-19 severity or susceptibility? Exploring these connections in greater detail would enhance the impact of the findings.**

## Author Response 10

We thank the reviewer for this comment. We observed some differences in ENPEP expression in select tissues between age groups and considered possible reasons in the discussion. However, we feel that the main takeaway is that, overall, ENPEP expression does not convincingly trend either up or down with age across different tissues. The sex-related changes in expression were even more slight, and the point was emphasized in the Discussion with more clarity. At this stage, when a possible role for ENPEP/APA in SARS-CoV-2 is still primarily theoretical, it is difficult to consider how these slight changes in ENPEP expression with age and sex could influence COVID-19 outcomes.

We have modified/added the following new text to the Discussion (italics indicates old text when new text is a modification of an existing sentence):

- “While tissue samples did not demonstrate uniform changes in ENPEP expression with age, this is yet to be studied in microvasculature and specific cell populations of interest such as pericytes. *Sex did not appear to play a significant role in ENPEP tissue expression in any of the analyses we performed,* making it unlikely that the gene has a marked influence on the differences in cardiovascular health or disease susceptibility between sexes. Even in the tissues with statistically significant differences in ENPEP expression between sexes, the differences in expression were slight, with marked overlap between males and females.”

# Reviewer 1 Comment 11

**7. Discussion and Interpretation:**

**The discussion section could benefit from a more thorough comparison of the findings with existing literature on other known SARS-CoV-2 cofactors.**

## Author Response 11

Thank you for this suggestion. We have accordingly expanded the Discussion regarding research on the other SARS-CoV-2 cofactors that we included in our correlation analysis, briefly discussing likely mechanisms, noting shared functions as aminopeptidases, and highlighted the lack of study of ENPEP/APA as a a viral receptor.

We have modified/added the following new text to the Discussion (italics indicates old text when new text is a modification of an existing sentence):

- “*We discovered that out of other genes associated with SARS-CoV-2 (i.e., DPP4, TMPRSS2, NRP1, and CTSL)*, and ANPEP (encoding APN, the HCoV-229E receptor [(Yeager et al. 1992)](https://paperpile.com/c/iCWWPK/hgMa)), *ENPEP has the strongest overall correlation* with ACE2 when assessing its expression in all tissues (0.46) and in vasculature (0.49).”
- Research has uncovered many of the viral mechanisms for SARS-CoV-2, with TMPRSS2 acting as the main cofactor for the viral receptor ACE2, cleaving the viral spike protein resulting in membrane fusion [(Takeda 2022)](https://paperpile.com/c/iCWWPK/HgqM), NRP1 acting as a cofactor and possible secondary viral receptor in the absence of ACE2 [(Cantuti-Castelvetri et al. 2020; Mayi et al. 2021)](https://paperpile.com/c/iCWWPK/QNGm+Q4t5), and CTSL enabling endosomal SARS-CoV-2 entry into cells as an alternative to TMPRSS2 mediated membrane fusion [(Jackson et al. 2022)](https://paperpile.com/c/iCWWPK/n8LD). DPP4, the MERS-CoV receptor, is also suggested to influence SARS-CoV-2 cell entry, but the evidence for this is less direct, mainly based on similarities between MERS-CoV and SARS-CoV-2 [(Sebastián-Martín et al. 2022)](https://paperpile.com/c/iCWWPK/UgcP), several computational binding studies between DPP4 and SARS-CoV-2 spike protein with differing results [(Li et al. 2020; Vankadari and Wilce 2020; Cameron et al. 2021)](https://paperpile.com/c/iCWWPK/hpzY+TnbO+83Qh), and studies indicating DPP4 inhibitor drugs might have a postive effect on COVID-19 outcomes in certain patient groups [(Sebastián-Martín et al. 2022; Nag et al. 2023)](https://paperpile.com/c/iCWWPK/UgcP+TaCk). However, APA also being a zinc aminopeptidase like the known human coronavirus receptor APN [(Luan and Xu 2007; Yang et al. 2013)](https://paperpile.com/c/iCWWPK/dJHE+EXq1), ENPEP appears understudied as a coronavirus cofactor candidate, with no studies focusing specifically on possible interactions between ENPEP and SARS-CoV-2.
- “*Although ENPEP and ACE2 correlate almost perfectly in the small intestine at 0.97 and both proteins localize in the enterocyte brush border, the same level of correlation is not observed elsewhere on a tissue-wide scale,* with the kidney cortex exhibiting the second highest ENPEP-ACE2 correlation at 0.82.”

# Reviewer 1 Comment 12

**Additionally, the implications of the strong correlation between ENPEP and angiogenesis-related genes like NRP1 should be explored further.**

## Author Response 12

Thank you for the comment. We improved the beginning of the discussion by highlighting the other angiogenesis-related genes correlated with ENPEP, and restructured the following paragraphs to better convey the functional implications of ENPEP as a driver of angiogenesis. Additionally, we highlight a lack of research regarding the possible relationships between ENPEP and some of these highly correlated angiogenic genes such as NRP1.

We have modified/added the following new text to the Discussion (italics indicates old text when new text is a modification of an existing sentence):

- “Other genes strongly related to angiogenesis, such as ADGRL4 (adhesion G protein-coupled receptor L4) [(Favara et al. 2019)](https://paperpile.com/c/aBw5Qw/O51t), TFPI (tissue factor pathway inhibitor) [(Holroyd et al. 2011)](https://paperpile.com/c/aBw5Qw/eKao), and VEGFC (vascular endothelial growth factor) [(Witzenbichler et al. 1998)](https://paperpile.com/c/aBw5Qw/CkYK) also ranked among the most correlated genes, further linking ENPEP to angiogenesis.”
- “Strong connections to angiogenesis and other processes related to vascular development imply roles for ENPEP in pathological states such as cancer, cardiovascular disease, and tissue remodeling. Fittingly, ENPEP has been connected to a variety of cancers and is upregulated in colorectal and renal neoplasms [(Chuang et al. 2017; Blanco et al. 2014)](https://paperpile.com/c/iCWWPK/DdbV+cW6o). Prior research also suggests that the expression of ENPEP is upregulated in other pathologic processes. Schlingemann et al. used immunostaining to demonstrate that APA is primarily present in tissues undergoing vascular generation, with tumor samples and damaged tissues such as inflamed synovial and granulation tissue exhibiting much more prominent staining than healthy vascular tissue, implying upregulation in tissues undergoing angiogenesis [(Schlingemann et al. 1996)](https://paperpile.com/c/iCWWPK/xZGm). However, this upregulation is not universal. As an example, iron-deficient liver tumors which have been found to exhibit reduced APA levels [(Wu et al. 2023)](https://paperpile.com/c/iCWWPK/cGjY), likely reflecting the diversity of conditions like cancer.”
- “While these studies clearly demonstrate the angiogenic properties of APA, particularly under stresses such as hypoxia, the gene appears not to be strictly essential as demonstrated by the viability of APA-knockout mice [(Kubota et al. 2010)](https://paperpile.com/c/iCWWPK/9v2j). APA upregulation in disease likely contributes to the regeneration of damaged vasculature, but the effects are not necessarily positive for the organism. Murine studies investigating the role of APA in post myocardial infarction have demonstrated RAAS dysregulation as a result of increased APA activity [(Alghamri et al. 2014)](https://paperpile.com/c/iCWWPK/KzJ0). As further evidence, treatment of mice with a centrally-acting APA inhibitor (Firibastat) after myocardial infarction resulted in better preserved cardiac function compared to controls [(Boitard et al. 2019)](https://paperpile.com/c/iCWWPK/15Xi). There is little, if any, research conducted on the possible interactions between APA and some of the highly correlated angiogenic proteins such as NRP1 and VEGFC. Inquiry into these relationships might uncover new mechanisms underlying angiogenesis.”
- “As it does in myocardial infarction [(Alghamri et al. 2014)](https://paperpile.com/c/iCWWPK/KzJ0), *ENPEP could also negatively impact the balance of the RAAS during COVID-19, regardless of its potential as a coreceptor*.”

# Reviewer 1 Comment 13

**How might ENPEP contribute to the vascular complications often observed in severe COVID-19 cases?**

## Author Response 13

We thank the reviewer for raising that question. We address possible connections between vascular complications and ENPEP in our response and additions to the text for comments 2 and 5.

In the original submission we have discussed in the Discussion the likely mechanism how ENPEP/APA could adversely affect RAAS balance:

- “As it does in myocardial infarction [(Alghamri et al. 2014)](https://paperpile.com/c/iCWWPK/KzJ0), ENPEP could also negatively impact the balance of the RAAS during COVID-19, regardless of its potential as a coreceptor. While SARS-CoV-2 interferes with ACE2 production of the vasoprotective and anti-inflammatory peptide ANG1-7 from ANG2 [(Tanzadehpanah et al. 2023)](https://paperpile.com/c/aBw5Qw/3loZ), ENPEP is upregulated by inflammation [(Schlingemann et al. 1996)](https://paperpile.com/c/aBw5Qw/EoNC). ANG2 can then be preferentially cleaved to ANG3 by APA, which can result in the production of inflammatory cytokines such as interleukin 6 (IL-6) by binding AT1 receptors [(Alanazi and Clark 2019)](https://paperpile.com/c/aBw5Qw/A5RN) — an effect likely pronounced in the CNS, where proinflammatory AT1 is the preferred receptor [(Wright et al. 1996; Höhle et al. 1995)](https://paperpile.com/c/aBw5Qw/dQVq+oHpF).”

We also made an addition to the part of the Discussion describing the vascular expression pattern of ACE2 and ENPEP, to state the implications of APA possibly promoting pericyte SARS-CoV-2 entry more clearly.

- “*If SARS-CoV-2 interacts with APA it could help explain the vascular effects of COVID-19 since pericytes are a systemic reservoir of cells in which APA and ACE2 are more strongly correlated than many of the currently known cofactors.* This pericyte-targeted infection could promote the systemic vascular inflammation, capillary leakage, and blood-brain-barrier disruption discussed above.”

# Reviewer 1 Comment 14

**Conclusion:**

**Overall, this manuscript offers valuable insights into the potential role of ENPEP in SARS-CoV-2 infection. With minor revisions, particularly in the areas of methodological clarity and discussion of the biological significance of the findings, the manuscript has the potential to make a significant contribution to the field.**

## Author Response 14

We thank the reviewer again for the valuable comments for improving the manuscript.

# Reviewer 1 Comment 15

**I recommend to cite following in discussion:**

**1- J. Al-Awaida, W., Jawabrah Al Hourani, B., Swedan, S., Nimer, R., Alzoughool, F., J. Al-Ameer, H., ... & R. Hadi, N. (2021). Correlates of SARS-CoV-2 Variants on Deaths, Case Incidence and Case Fatality Ratio among the Continents for the Period of 1 December 2020 to 15 March 2021. Genes, 12(7), 1061.**

**2- Khirfan, F., Jarrar, Y., Al-Qirim, T., Goh, K. W., Jarrar, Q., Ardianto, C., ... & Ming, L. C. (2022). Analgesics induce alterations in the expression of SARS-CoV-2 entry and arachidonic-acid-metabolizing genes in the mouse lungs. Pharmaceuticals, 15(6), 696.**

**3- Hatmal, M. M. M., Al-Hatamleh, M. A., Olaimat, A. N., Mohamud, R., Fawaz, M., Kateeb, E. T., ... & Bindayna, K. M. (2022). Reported adverse effects and attitudes among Arab populations following COVID-19 vaccination: a large-scale multinational study implementing machine learning tools in predicting post-vaccination adverse effects based on predisposing factors. Vaccines, 10(3), 366.**

## Author Response 15

Thank you for the recommended reading. We found the second article to be of most relevance for the work that we have completed.

We have modified/added the following new text to the Discussion (italics indicates old text when new text is a modification of an existing sentence):

- “*ANG2 can then be preferentially cleaved to ANG3 by APA, which can result in the production of inflammatory cytokines such as interleukin 6 (IL-6) by binding AT1 receptors* [*(89)*](https://paperpile.com/c/fMVWwv/PtbA) *— an effect likely pronounced in the CNS, where proinflammatory AT1 is the preferred receptor* [*(90,91)*](https://paperpile.com/c/fMVWwv/9rEq+WHmf)*.* A study of NSAID treatment in mice has shown that ACE2 is strongly downregulated and TMPRSS2 upregulated in the lung after NSAID treatment [(Khirfan et al. 2022)](https://paperpile.com/c/aBw5Qw/PxJ4) with similar changes observed in rat kidney [(Saheb Sharif-Askari et al. 2020)](https://paperpile.com/c/aBw5Qw/hgwO), supporting the idea that inflammation-associated changes could drive dysregulation of RAAS and impact cell infection rates.”
